# Prevention and Control of COVID-19 in Chronic Kidney Disease

**DOI:** 10.1007/s12098-020-03306-y

**Published:** 2020-04-28

**Authors:** Shipin Feng, Min Xie, Wei Luo, Li Wang, Limin Guo, Ying Wu, Jun Liu, Qinwei Duan, Qin Yang, Jia Li, Xi Liu, Rong Zhu

**Affiliations:** Department of Pediatric Nephrology, Chengdu Women’s and Children’s Central Hospital, Chengdu, Sichuan China

*To the Editor:* Since the outbreak of COVID-19 in Wuhan, China, the epidemic caused by COVID-19 has become a public health event of international concern, which is seriously threatening the health of people worldwide. As is known, children with chronic kidney disease **(**CKD) who are treated with long-term glucocorticoids and immunosuppressants belong to the high-risk and susceptible group of the 2019- novel coronavirus (2019-nCoV). However, up to date, there is no case report of CKD complicated with COVID-19 in China. We here present our experience in the prevention and control of COVID-19 infection in this special group of patients, with a purpose to provide reference for the international clinical community. The general management comprises of proper rest, adequate nutrition support, and symptomatic treatment. For antiviral therapy, α - interferon, lopinavir / ritonavir, or abidol can be used.

Glucocorticoids in mild and common type COVID-19: For CKD children in the consolidation and maintenance stage of hormone treatment, the frequency of oral hormone use can be changed from every other day to every day for 7 consecutive days at the same dose in order to reduce the recurrence of the primary disease [1]; for those in the stage of hormone-induced remission, the dose of hormone can be tapered according to the patient’s condition.

Glucocorticoids in COVID-19 severe and critical type: Hormone dosage should be adjusted according to the degree of systemic inflammatory reaction and whether the patient is complicated with acute respiratory distress syndrome (ARDS). It is recommended not to exceed 1–2 mg/kg per day used for methylprednisolone.

Immunosuppressants: Cyclosporine and mycophenolate mofetil were found to significantly inhibit the replication of Middle East respiratory syndrome coronavirus (MERS CoV) and severe acute respiratory syndrome coronavirus (SARS CoV) [2]. However, there is no report about the effect of these immunosuppressants on 2019-nCoV up-to-date. Therefore, the use of immunosuppressants in children with CKD should be determined according to the specific condition of the patient.

Management of blood pressure: The pathogenesis of 2019-nCoV is similar to that of SARS virus [3], which infects human cells by recognizing and binding to angiotensin-converting enzyme (ACE)-2 protein; the use of ACE inhibitors (ACEIs) and angiotensin receptor antagonists (ARBs) can increase the expression and activity of ACE-2 [4]. Therefore, it is suggested that ACEIs and ARBs should be discontinued in children with COVID-19 and other antihypertensive drugs should be used instead.
